# Prelamin A impairs 53BP1 nuclear entry by mislocalizing NUP153 and disrupting the Ran gradient

**DOI:** 10.1111/acel.12506

**Published:** 2016-07-27

**Authors:** Andrew M. Cobb, Delphine Larrieu, Derek T. Warren, Yiwen Liu, Sonal Srivastava, Andrew J. O. Smith, Richard P. Bowater, Stephen P. Jackson, Catherine M. Shanahan

**Affiliations:** ^1^ The James Black CentreKing's College London125 Coldharbour LaneLondonSE5 9NUUK; ^2^ Wellcome Trust/Cancer Research UK Gurdon InstituteThe Henry Wellcome Building of Cancer and Developmental BiologyUniversity of CambridgeTennis Court RoadCambridgeCB2 1QNUK; ^3^ School of Biological SciencesUniversity of East AngliaNorwichNR4 7TJUK

**Keywords:** 53BP1, cytoplasmic–nuclear trafficking, NUP153, prelamin A, Ran gradient, vascular disease

## Abstract

The nuclear lamina is essential for the proper structure and organization of the nucleus. Deregulation of A‐type lamins can compromise genomic stability, alter chromatin organization and cause premature vascular aging. Here, we show that accumulation of the lamin A precursor, prelamin A, inhibits 53BP1 recruitment to sites of DNA damage and increases basal levels of DNA damage in aged vascular smooth muscle cells. We identify that this genome instability arises through defective nuclear import of 53BP1 as a consequence of abnormal topological arrangement of nucleoporin NUP153. We show for the first time that this nucleoporin is important for the nuclear localization of Ran and that the deregulated Ran gradient is likely to be compromising the nuclear import of 53BP1. Importantly, many of the defects associated with prelamin A expression were significantly reduced upon treatment with Remodelin, a small molecule recently reported to reverse deficiencies associated with abnormal nuclear lamina.

## Introduction

Lamins are intermediate filament proteins that assemble into structures forming the nuclear lamina. This platform underlies the inner nuclear membrane (INM) and provides mechanical stability to the nucleus (Gruenbaum *et al*., 2005) as well as influencing several DNA metabolism events including the organization of chromatin, transcription and DNA repair (Dittmer & Misteli, 2011; Warren & Shanahan, 2011). The nuclear lamina is comprised of A‐type (A and C) and B‐type (B_1_ and B_2_) lamins, which exhibit specific differences in biochemical properties. Lamin A requires several enzymatic processing events for maturation from its precursor prelamin A to mature lamin A, with the final step being the endoproteolytic removal of a C‐terminal farnesylated cysteine residue by Zmpste24/Face1 (Davies *et al*., 2011). It is hypothesized that the role of the hydrophobic farnesyl and carboxymethyl moieties attached to C‐terminal cysteines of some lamin molecules is to anchor them to the INM lipid bilayer (Barrowman *et al*., 2012), thereby influencing localization and function.

At least 11 distinct human diseases have been associated with >300 mutations in the gene that codes for lamins A and C (*LMNA*) (Dechat *et al*., 2008), including the premature aging disease Hutchinson–Gilford progeria syndrome (HGPS) (Coutinho *et al*., 2009; Rodriguez *et al*., 2009). Fibroblasts taken from individuals with HGPS exhibit defective lamin A processing and accumulate a permanently farnesylated, truncated form of prelamin A termed progerin that induces abnormal nuclear morphologies and elevated levels of DNA damage (Liu *et al*., 2005) that are thought to contribute to disease pathologies (McClintock *et al*., 2006). The main pathological feature of HGPS is vascular smooth muscle cell (VSMC) degeneration and calcification (Salamat *et al*., 2010). Because VSMCs function as major components of the vessel wall and are responsible for maintaining vascular tone and mediating vessel repair (Warren & Shanahan, 2011), their ablation in HGPS leads to death by accelerated arteriosclerosis, leading to myocardial infarction or stroke (Denecke *et al*., 2006; Merideth *et al*., 2008).

Importantly, studies have shown that prelamin A may also contribute to vascular degeneration in atherosclerotic patients and aged individuals in the general population (Lattanzi *et al*., 2007; Ragnauth *et al*., 2010; Warren & Shanahan, 2011; Mahen *et al*., 2013). VSMCs from normal individuals naturally accumulate prelamin A *in vitro* and *in vivo* prior to undergoing senescence, and therefore, prelamin A can be used as a biomarker of vascular aging (Ragnauth *et al*., 2010; Zhang *et al*., 2011). Its expression is linked to VSMC dysfunction, pathological changes in gene expression that promote vascular calcification and elevated levels of DNA damage (Liu *et al*., 2013).

The DNA damage response (DDR) is a tightly coordinated succession of events that orchestrates the detection, signalling and removal of DNA lesions. Studies have shown that increased DNA damage in HGPS cells is due in part to delays in the formation of DNA damage foci (Liu *et al*., 2005) and reduced stability of p53‐binding protein 1 (53BP1) (Gonzalez‐Suarez *et al*., 2009). This protein is a key component in the repair of double‐strand breaks (DSBs) that are a highly toxic form of DNA lesion. In mammalian cells, DSBs are detected by surveillance proteins that lead to the phosphorylation of histone variant H2AX, forming a phosphorylated species termed γH2AX within microscopically discernible foci (Rogakou *et al*., 1998). This then leads to localized ubiquitination of γH2AX that promotes recruitment of 53BP1, which dictates how the break is repaired (Mailand *et al*., 2007; Goodarzi & Jeggo, 2013). 53BP1 recruitment depends upon its import into the nucleus and subsequent targeting to DNA damage by recognizing histone modifications (Panier & Boulton, 2014).

In this study, we present evidence that in aged VSMCs, prelamin A interrupts the nuclear import of 53BP1 leading to its cytoplasmic accumulation by causing spatial deregulation of the nucleoporin NUP153 and subsequent interference of the Ran gradient. We also show that unlike farnesyltransferase inhibitors (FTIs), which showed little effect on reversing NUP153 deregulation, the small molecule Remodelin appeared to alleviate 53BP1 cytoplasmic accumulation, reduce genomic instability, restore nuclear circularity and delay senescence in aged VSMCs.

## Results

### Prelamin A inhibits 53BP1 recruitment to DNA damage in aged VSMCs by inducing cytoplasmic accumulation

**Figure 1:**
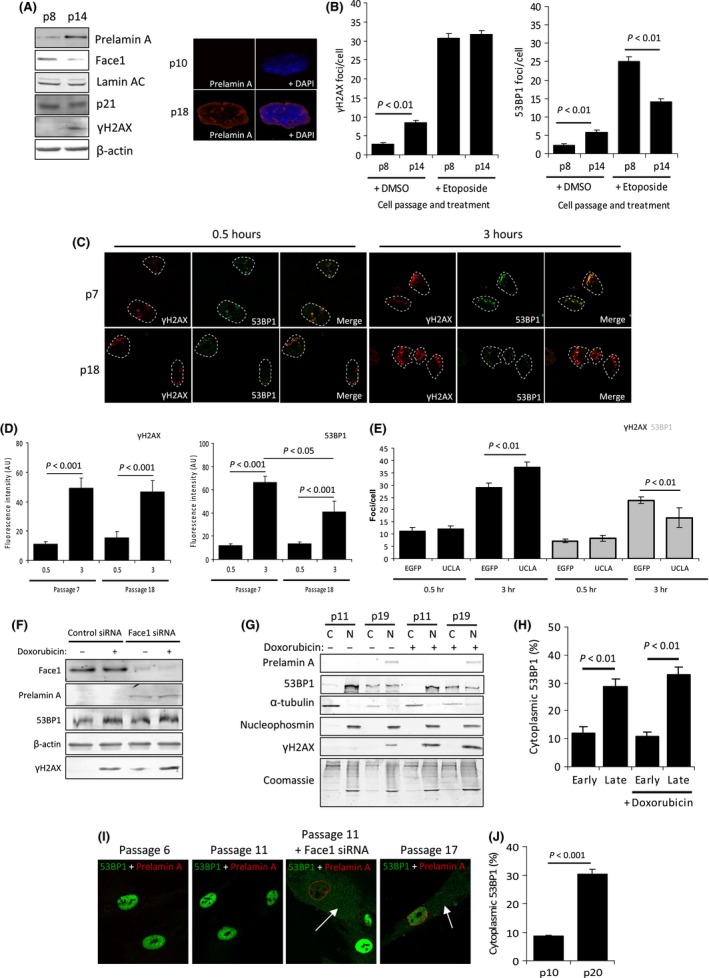
Prelamin A in aged VSMCs prevents 53BP1 recruitment to DNA damage by inducing cytoplasmic accumulation. (A) (Left) WB showing increased prelamin A in aged (p14) VSMCs compared to early (p8) VSMCs that occurs concomitantly with a decrease in Face1. Levels of mature lamin AC and p21 are not markedly different, whereas γH2AX is increased. The data shown are from 35F VSMC isolate, but we also detect prelamin A accumulation and increased γH2AX in two other VSMC isolates (Fig. S1). All experiments were repeated a minimum of 3 times. (Right) IF image of a proliferative early passage (p10) VSMC and a nonproliferative late passage (p18) VSMC stained for prelamin A (red) and DAPI (blue). (B) Enumeration of γH2AX (left) and 53BP1 (right) foci in p8 and p14 VSMCs treated with DMSO or etoposide. *n *>* *200 cells per treatment taken from 3 independent experiments. Standard errors are shown. (C) IF showing γH2AX (red) and 53BP1 (green) in p7 and p18 VSMCs treated with microirradiation and left to recover for 0.5 or 3 h. About 84% of p18 VSMCs were positive for prelamin A, and no prelamin A was detected in p7 VSMCs (data not shown). (D) Quantification of C. *n *>* *100 cells per treatment taken from 3 independent experiments. (E) IF analysis of γH2AX and 53BP1 foci in p8 control (EGFP) or expressing prelamin A (UCLA) VSMCs treated with etoposide for 3 h. *n *=* *200 cells per treatment taken from 3 independent experiments. (F) Whole cell lysate WB taken from early passage VSMCs treated with control or Face1 siRNA and −/+ doxorubicin treatment. Levels of prelamin A were increased, but total 53BP1 protein level did not change. (G) WB of cell fractionation analysis showing cytoplasmic 53BP1 accumulates in p19 VSMCs and that this coincides with prelamin A expression (not seen in p11 VSMCs). α‐tubulin and nucleophosmin are shown as controls for cytoplasmic (C) and nuclear (N) fractions, respectively. Doxorubicin did not affect this accumulation. (H) Quantification of G. Data were taken from a minimum of 3 separate experiments. (I) IF of 53BP1 (green) cytoplasmic accumulation in VSMCs that have accumulated prelamin A (red). p6 and p11 VSMCs had undetectable levels of prelamin A, but induced expression of Prelamin A with Face1 siRNA (third panel) caused an increase in levels of cytoplasmic 53BP1 and reduced nuclear levels. This change is also evident in p17 VSMCs that have naturally accumulated prelamin A. White arrows indicate cytoplasmic 53BP1. Nuclei have been stained with DAPI. (J) Quantification of fluorescence measurements of cytoplasmic and nuclear 53BP1 in p10 and p20 VSMCs. *n *>* *100 cells from 3 separate experiments.

Human VSMCs accrue prelamin A during aging (Ragnauth *et al*., 2010), and this can be replicated *in vitro* by serial passaging (Fig. 1A and Fig. S1). To determine whether aged VSMCs display defects in the DDR, we examined their response to DNA damage. Late passage (p14) VSMCs with acquired prelamin A accumulation exhibited higher basal levels of γH2AX and 53BP1 foci than proliferating early passage (p8) VSMCs. Etoposide treatment increased γH2AX staining in both cell populations; however, there were significantly less 53BP1 foci in p14 compared to p8 VSMCs (Fig. 1B). To explore the basis for attenuated 53BP1 recruitment, we assessed H2AX and 53BP1 foci formation at sites of DNA damage induced by laser microirradiation in early and late passage VSMCs. We found that 3 h after irradiation, despite unaffected γH2AX formation, the late passage VSMCs displayed reduced 53BP1 accumulation at laser lines (Fig. 1C,D). No differences in 53BP1 were seen 0.5 h after damage induction implying that immediately after DNA damage induction, initial recruitment of 53BP1 is normal in these cells. To establish whether the decrease in 53BP1 foci formation was directly caused by prelamin A, we expressed an uncleavable form of lamin A (UCLA) in passage 8 VSMCs, induced DNA damage, then compared γH2AX and 53BP1 foci formation to controls expressing EGFP (Fig. 1E). Again, despite an increase in γH2AX, 53BP1 was significantly reduced by prelamin A expression. To test whether overexpression of wild‐type lamin A also affected 53BP1 recruitment to DNA damage, we expressed EGFP, wild‐type lamin A and UCLA in VSMCs and only found prelamin A‐expressing cells exhibited reduced 53BP1 at DNA lesions (Fig. S2). In parallel experiments to the above, we observed similar defects in U2OS cells expressing prelamin A, including nuclear blebbing and attenuated 53BP1 recruitment (Fig. S3).

Next, we investigated how prelamin A might affect 53BP1 recruitment to DNA damage sites. Firstly, we assessed whether 53BP1 protein stability was affected as deregulated nuclear lamin has been previously implicated in reducing stability of this protein (Gonzalez‐Suarez *et al*., 2011). Western blots detected no changes in cellular levels of 53BP1 either in VSMCs expressing prelamin A through Zmpste24/Face1 depletion, or in the presence of DSBs induced with doxorubicin (Fig. 1F). We also examined whether methylation of histones H3K79 and H4K20, which are known to contribute to 53BP1 recruitment, was affected. Analysis of protein levels by Western blot and also spatial organization by immunofluorescence of both species showed no differences between cells with or without prelamin A expression, or upon DNA damage induction (Fig. S4).

Analysis of the subcellular localization of 53BP1 in VSMCs using Western blot of cytoplasmic and nuclear fractions (Fig. 1G,H) established that cytoplasmic 53BP1 was more abundant in late passage VSMCs than in early passage VSMCs, with a statistically significant shift of 53BP1 to the cytoplasm from the nucleus during passaging that was independent of DNA damage. These changes were also observable by immunofluorescence microscopy, either in aged or in young VSMCs depleted of Face1 to accumulate prelamin A (Fig. 1I,J). To verify that prelamin A‐induced cytoplasmic accumulation of 53BP1 was not cell type specific, we also analysed U2OS cells that were expressing UCLA by biochemical cell fractionation and noted similar accumulation of 53BP1 in the cytoplasm (Fig. S5).

### NUP153 mislocalization by prelamin A restricts 53BP1 nuclear entry

**Figure 2:**
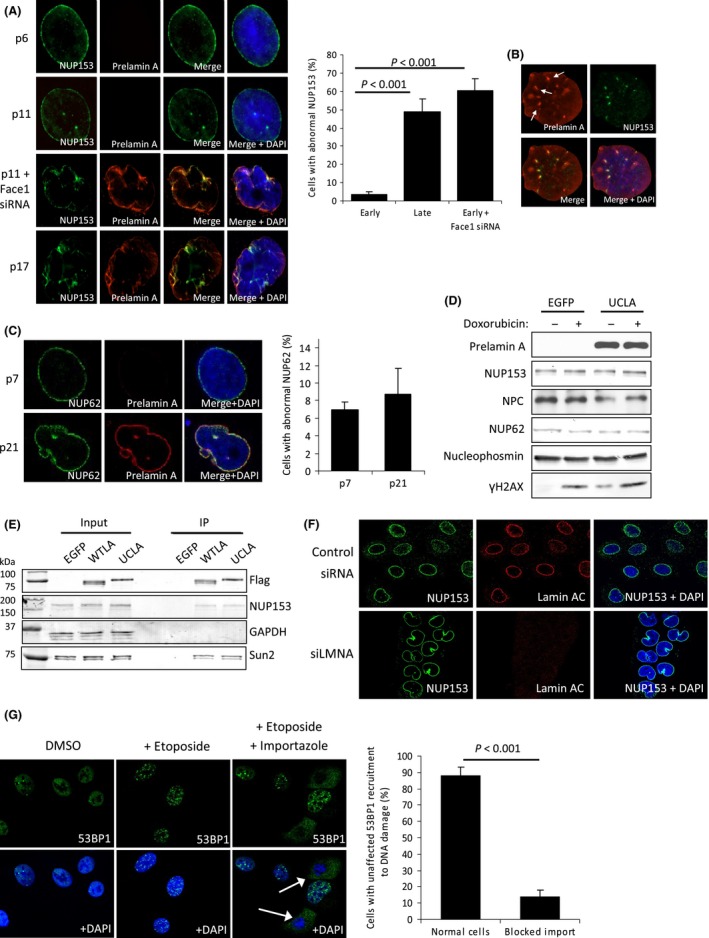
Prelamin A accumulation in aged VSMCs induces NUP153 mislocalization. (A) (Left) IF of NUP153 (green) and prelamin A (red) in passages 6, 11, 11 + Face1 siRNA and 17. DNA is stained with DAPI. Prelamin A results in nucleoplasmic aggregation of NUP153 and its loss at the NE. (Right) Quantification of incidence of abnormal NUP153. *n *>* *300 cells from 3 separate experiments, standard errors are shown. (B) IF of a p11 VSMC nuclei that was induced to express Flag‐tagged prelamin A. NUP153 (green) and prelamin A (red) colocalization is shown by white arrows. DNA is stained with DAPI. (C) (Left) IF analysis of NUP62 (green) in p7 and p21 VSMCs. Despite prelamin A‐induced deformation of the nuclei, most NUP62 is retained at the NE. DAPI is shown. (Right) Quantitative analysis of cells displaying abnormal NUP153, *n *>* *200 cells from 3 independent experiments. (D) WB of nucleoporin protein levels in early passage VSMCs expressing EGFP or prelamin A (UCLA). No marked differences were observed. (E) WB showing example from Flag precipitation experiments used to assess NUP153 interactions with mature wild‐type lamin A (WTLA) and prelamin A (UCLA). EGFP was used as a negative control. Assays revealed NUP153 was precipitated when either WTLA or UCLA were used as bait. (F) IF showing depletion of lamin AC (red) using siLMNA induces changes in nuclear morphology but does not cause loss of NUP153 (green) from the NE. DNA is stained with DAPI (blue). (G) (Left) IF of cells treated with Importazole for 24 h to block importin‐β‐mediated import of 53BP1 (green). Cells exhibited attenuation of nuclear 53BP1 foci after etoposide treatment, reinforcing the importance of nuclear import of 53BP1 for its activity. (Right) Quantification of cells treated with etoposide and Importazole. Foci formation of 53BP1 was compared in cells that displayed no cytoplasmic 53BP1 accumulation (normal cells) and those showing increased cytoplasmic 53BP1 (blocked import). Cells with >5 53BP1 foci were considered to have unaffected 53BP1 recruitment. *n *>* *150 cells from 3 separate experiments, standard errors are shown.

Nuclear import of 53BP1 is in part mediated by nucleoporin NUP153 (Lemaitre *et al*., 2012; Moudry *et al*., 2012). The nuclear lamina is essential for the positioning of nuclear pore complexes (NPCs), and NUP153 has been shown to bind directly to both A‐ and B‐type lamins (Al‐Haboubi *et al*., 2011). Importantly, prelamin A has previously been implicated in the disorganization of NUP153 (Goulbourne *et al*., 2011) and immunofluorescence revealed a reduction of NUP153 on the nuclear surface and an increase in intranuclear clustering in late passage VSMCs (Fig. 2A). Furthermore, we found that depletion of Face1 in early passage VSMCs (that previously displayed normal NUP153 localization) induced defects even more marked than those observed in late passage cells. In both instances, the characteristic nuclear envelope (NE) staining of NUP153 appeared diminished with the appearance of intranuclear aggregations that consistently colocalized with prelamin A (Fig. 2B). These foci have been reported to be invaginations caused by folding of either the INM or both the INM and outer nuclear membrane (Goulbourne *et al*., 2011). However, not all nucleoporins displayed such perturbed positioning upon prelamin A expression as evident by NUP62 localization (Fig. 2C), suggesting that prelamin A expression does not impair overt NPC assembly and localization. Western blot analysis of a range of nucleoporins including NUP153 following UCLA expression or treatment with doxorubicin revealed prelamin A in early passage VSMCs had no discernible effect on the levels of these proteins (Fig. 2D), implying that the observed decrease in NUP153 at the NE was caused by changes in its localization and not loss of protein.

To elucidate whether prelamin A affected positioning of NUP153 via direct interactions, we performed co‐immunoprecipitation experiments in U2OS cells expressing flag‐epitope‐tagged prelamin A (UCLA), wild‐type mature lamin A (WTLA) or EGFP control. Analysis of coprecipitated proteins revealed that NUP153 interacted with both mature lamin A and prelamin A (Fig. 2E), indicating prelamin A may act as a binding competitor against mature lamin A for NUP153 and thereby alter NUP153 spatial arrangement. The confirmation that mature lamin A interacted with NUP153 led us to question whether its depletion would cause similar phenotypes to that of prelamin A accumulation. In contrast, siRNAs against *LMNA* did not induce trapping of NUP153 into NR or reduce its localization at the ER (Fig 2F).

To further understand the impact of reduced import of 53BP1 into the nucleus following NUP153 mislocalization, we inhibited importin‐β‐mediated import in VSMCs using Importazole, induced DNA damage and determined 53BP1 foci formation by immunofluorescence. As shown in Fig. 2G, cells exhibiting restricted nuclear entry of 53BP1 following Importazole treatment (white arrows) had attenuated foci formation at DSBs 3 h after etoposide treatment. This result highlights the importance of unimpeded trafficking of 53BP1 between nucleus and cytoplasm for it to function during DNA repair.

### Disruption of NUP153 deregulates Ran localization

**Figure 3:**
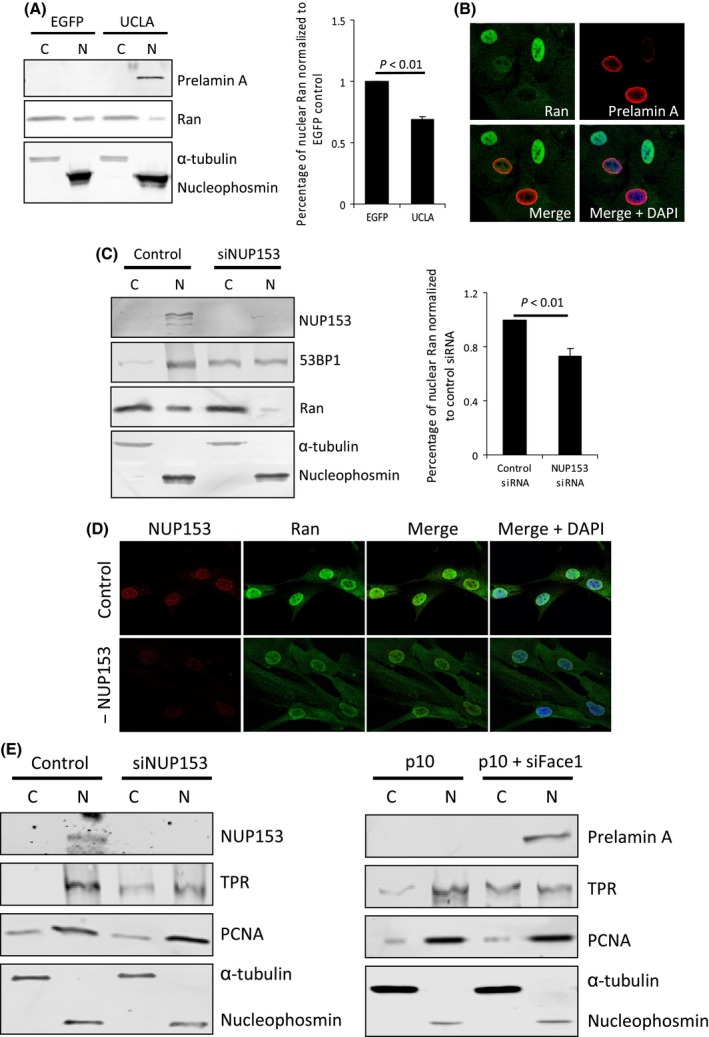
The Ran gradient is disrupted by prelamin A expression or depletion of NUP153. (A) (Left) WB of p10 VSMC cytoplasmic (C) and nuclear (N) fractions following EGFP or UCLA expression. α‐tubulin (cytoplasmic) and nucleophosmin (nuclear) are shown as loading controls. Quantification of Ran from 3 independent experiments (standard errors are shown) (Right). (B) IF image of Ran (green) in p10 VSMCs expressing EGFP or UCLA. Prelamin A (red) and DAPI (blue) are shown. (C) (Left) WB of p10 VSMC cytoplasmic (C) and nuclear (N) fractions following control or NUP153 siRNA treatment and ensuing cytoplasmic accumulation of 53BP1. α‐tubulin (cytoplasmic) and nucleophosmin (nuclear) are shown as loading controls. (Right) Quantification of Ran from 3 independent experiments. (D) IF of Ran (green) in p10 VSMCs treated with control or NUP153 siRNA. DAPI is shown in blue. (E) (Left) WB showing NUP153 depletion can affect nuclear localization of TPR but not PCNA in p9 VSMCs. (Right) WB showing Face1 depletion in p10 VSMCs induces similar cytoplasmic accumulation of TPR.

Previous studies (Kelley *et al*., 2011; Snow *et al*., 2013) highlighted a role for progerin in the abrogation of the Ran protein gradient. Therefore, we tested whether prelamin A caused a similar phenotype and whether this was caused by mislocalization of NUP153. We expressed UCLA in low passage VSMCs to ‘age’ them and then performed nuclear fractionations of these and EGFP‐transduced control cells. Western blot and densitometry (Fig. 3A) showed that prelamin A did decrease nuclear Ran and this was corroborated by immunofluorescence showing VSMCs positive for prelamin A displayed lower nuclear Ran and higher cytoplasmic Ran (Fig. 3B). Our next aim was to establish whether mislocalization of NUP153 was influencing this defect; therefore, we used siRNA to deplete NUP153 in early passage VSMCs and used nuclear fractionation (Fig. 3C) and immunofluorescence (Fig. 3D) to analyse Ran localization. As before, we detected reduced nuclear levels and increased cytoplasmic levels, suggesting that mislocalization of NUP153 by prelamin A is disrupting the Ran protein gradient preventing import of 53BP1. To further support the finding that NUP153 mislocalization was affecting protein import by changes to the Ran gradient, we used cell fractionation to test the importin‐β‐mediated nuclear import of a large cargo (TPR) and a relatively smaller cargo (PCNA) in cells depleted in NUP153 (Fig. 3E). We observed increased cytoplasmic levels of TPR but not PCNA, indicating that larger cargos are most affected by defects in the Ran gradient as previously described (Snow *et al*., 2013). As NUP153 mislocalization affects nuclear import of larger cargo, we hypothesized that TPR would also show impeded nuclear entry in VSMCs expressing prelamin A. We used Western blot to analyse cell fractions from control p10 VSMCs or p10 VSMCs depleted of Face1 to determine cellular localization of TPR and the smaller PCNA. We found TPR cytoplasmic accumulation, but not PCNA cytoplasmic accumulation (Fig. 3E), thus supporting our hypothesis that large cargo nuclear import is affected in VSMCs expressing prelamin A.

### Farnesyltransferase inhibitors only modestly restore NUP153 positioning and do not alleviate prelamin A toxicity in aged VSMCs

**Figure 4:**
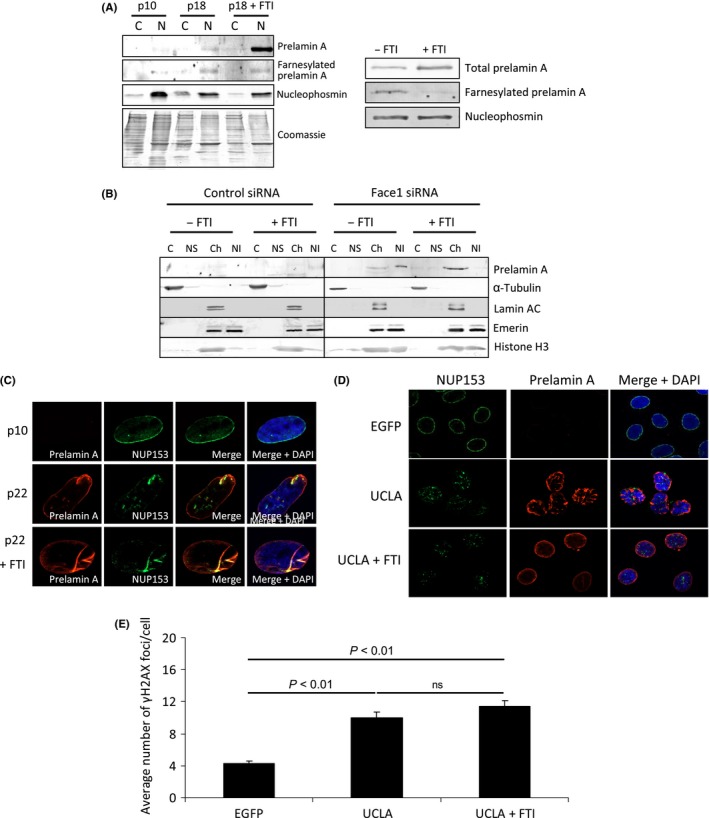
FTIs improve prelamin A‐induced dysmorphic nuclei but do not recover NUP153 localization or genome stability. (A) (Left) WB showing prelamin A that accumulates naturally in aged VSMCs (p18) is farnesylated using an antibody specific for farnesylated prelamin A. Cells were fractionated into cytoplasmic (C) and nuclear (N) compartments to concentrate proteins. FTIs significantly increase levels of total prelamin A, but this increase is not detected with the farnesylated prelamin A‐specific antibody. (Right) WB of U2OS cells expressing UCLA. Cells had been treated with or without FTI‐276 for 48 h prior to harvesting. Protein was probed using antibodies against total prelamin A or specific for farnesylated prelamin A. (B) WB of biochemical fractionated U2OS cells treated with control or Face1 siRNA and −/+ FTIs. C – cytoplasmic, NS – nuclear soluble, Ch – chromatin and NI – nuclear insoluble. Prelamin A is tightly associated with the NE and remains in the nuclear fraction unlike the more soluble lamin A/C. FTIs release prelamin A from the insoluble fraction. (C) IF of NUP153 (green) localization in p10 and p22 VSMCs −/+ FTIs. Prelamin A (red) and DNA (blue) are also shown. (D) IF showing NUP153 (green) and prelamin A (red) in U2OS cells expressing either EGFP or UCLA −/+ FTIs. DAPI (blue) is also shown. (E) Enumeration of γH2AX in VSMCs expressing EGFP, UCLA alone or UCLA + FTI treatment. *n *>* *200 cells were analysed from 3 independent experiments, and average number of γH2AX foci per cell was calculated. Standard errors are shown.

The retention of the hydrophobic farnesyl moiety on prelamin A has been proposed to be a primary factor in its deleterious effects, potentially by enhanced anchoring to the NE (Wang *et al*., 2012). Using specific antibodies, farnesylated prelamin A was detected in aged VSMCs and in U2OS cells expressing UCLA (Fig. 4A left and right, respectively). Biochemical fractionation showed that prelamin A was mainly in the nuclear insoluble fraction, while mature lamins A and C were present in the chromatin‐associated fraction (Fig. 4B) consistent with the notion that interactions between mature lamins A and C and the NE are weaker than those of prelamin A. Furthermore, treating cells with the farnesyl transferase inhibitor FTI‐276 caused prelamin A to elute in the chromatin fraction, reinforcing a model in which the farnesyl group plays a key role in anchoring prelamin A to the NE.

FTIs restore nuclear shape in progerin‐ or prelamin A‐positive cells, so our next aim was to determine whether NUP153 localization could be normalized by FTI exposure. Notably, although treatment of prelamin A‐positive cells with FTI‐276 was able to restore normal nuclear shape, there was only limited restoration in NUP153 positioning around the NE (Fig. 4C and D). As previously found (Liu *et al*., 2006; Larrieu *et al*., 2014), FTI treatment had no effect on basal DNA damage levels (Fig. 4E), indicating that the farnesyl group of prelamin A is not wholly responsible for the toxicity of this protein, or that once farnesylated prelamin A has been present in cells, the subsequent removal of the farnesyl group is insufficient to restore nuclear homeostasis.

### Remodelin treatment alleviates defects associated with prelamin A accumulation

**Figure 5:**
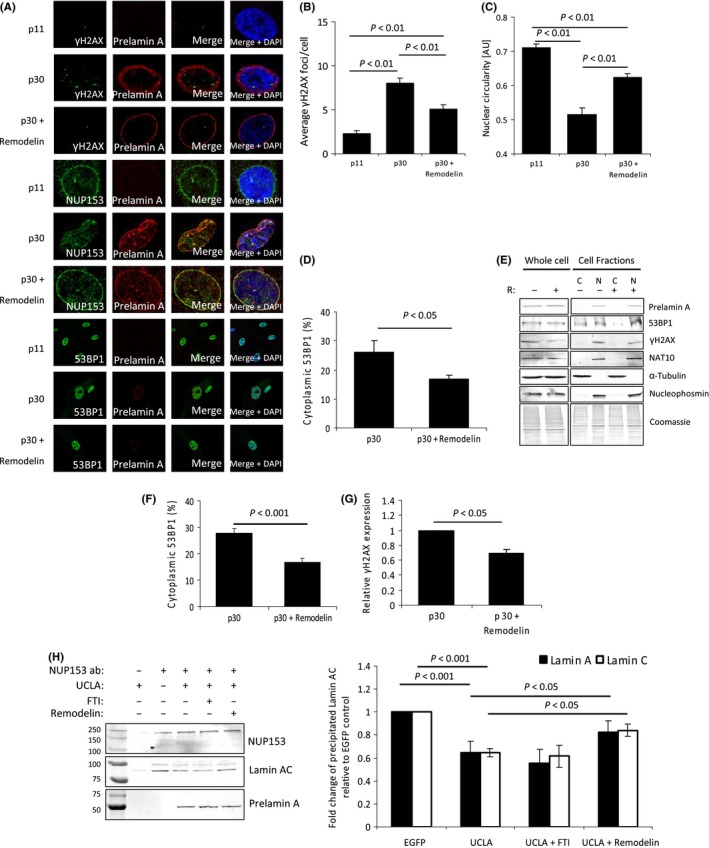
Remodelin reverses prelamin A‐dependent defects in aged VSMCs. (A) IF of p11, p30 and p30 +Remodelin VSMCs. Cells were stained for γH2AX, NUP153 and 53BP1 (all green) in addition to prelamin A (red) and DAPI (blue). Panels showing 53BP1 staining show cells at a reduced magnification to allow visualization of staining in cell cytoplasmic regions. (B) Quantification of average number of γH2AX in p11, p30 and p30 + Remodelin VSMCs. *n *>* *100 cells were counted per cell group from 3 independent experiments. Standard errors are shown. (C) Nuclear circularity of p11, p30 and p30 + Remodelin VSMCs. Values closer to 1 indicate nuclei that are more circular. *n *>* *100 cells from more than 3 independent experiments. (D) Fluorescence intensity measurements of cytoplasmic 53BP1 in p30 and p30 + Remodelin VSMCs. Readings for nuclear and cytoplasmic were obtained, and percentage cytoplasmic values were calculated. *n *>* *100 cells from 3 independent experiments. (E) WB of whole cell and cell fractions (cytoplasmic – C, nuclear – N) of p30 VSMCs −/+ Remodelin (R). (F) Quantification of 53BP1 bands shown in D. Data are from 3 separate experiments. (G) Quantification of γH2AX bands shown in D. Data are from 3 separate experiments (H) (Left) Co‐immunoprecipitation WB using NUP153 as bait with lamin AC and prelamin A as target interactors. Assays were performed in U2OS cells expressing UCLA −/+FTIs and −/+ Remodelin. Prelamin A acted as a competitor against lamin AC to bind to NUP153, but this was alleviated to an extent by addition of Remodelin. (Right) Quantification of lamin A and C precipitation. Band intensities of precipitated lamin AC were measured and normalized to total input lamin AC. Fold changes of precipitated lamin AC in UCLA, UCLA + FTI and UCLA + Remodelin were then calculated relative to EGFP control cells. *n *=* *4, standard errors are shown.

The recent discovery that an inhibitor of the N‐acetyl‐transferase NAT10, termed Remodelin (Larrieu *et al*., 2014), was able to reverse cellular deficiencies in both HGPS fibroblasts and lamin A/C depleted cells led us to test whether prelamin A‐induced aberrations in aged VSMCs could be alleviated by Remodelin. Immunofluorescence analysis showed late passage VSMCs treated with Remodelin had significantly less γH2AX compared to untreated cells (Fig. 5A,B), although it did not return to levels observed in early passage VSMCs. It was also evident that NUP153 localization at the NE was markedly improved alongside restored nuclear circularity (Fig. 5C) and reduced cytoplasmic 53BP1 (Fig. 5A,D). Nuclear fractionation also showed decreased levels of cytoplasmic 53BP1 and reduced γH2AX levels (Fig. 5E–G) despite no observable changes in prelamin A or NAT10 levels. In addition, we found Remodelin was able to improve nuclear morphology, improve genomic stability and restore 53BP1 nuclear compartmentalization in U2OS cells expressing prelamin A (Fig. S6).

As we had shown prelamin A could interact with NUP153 (Fig. 2E) and this was likely to be a key factor in its mislocalization and reduced functionality, we wondered if the improvements identified by Remodelin treatment had affected these interactions. We used NUP153 as bait protein and assessed co‐immunoprecipitation of lamin AC and prelamin A (Fig. 5H). As predicted, both mature lamins A and C were found to interact with NUP153 and upon expression of UCLA, this was decreased, with prelamin A being precipitated instead. FTI treatment had minimal effect upon this; however, Remodelin did improve NUP153 interactions with mature lamins despite not preventing prelamin A‐NUP153 interactions. It is unclear how NUP153 was able to accommodate binding to both prelamin A and lamins AC, but the improved interaction with the mature lamina following Remodelin treatment may explain NUP153 localization improvements.

### Remodelin improves cell health and genomic instability of aged VSMCs

**Figure 6:**
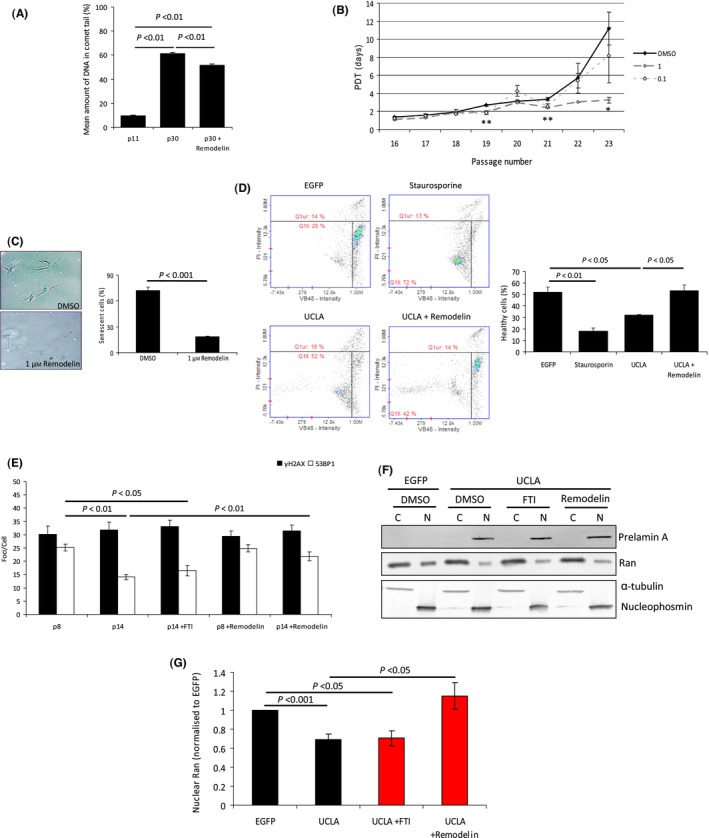
Remodelin improves cell fitness of late passage VSMCs and improves Ran gradient defects associated with prelamin A expression. (A) Comet assay of p12, p30 and p30 + Remodelin VSMCs. *n *>* *100 cells per group were analysed from 4 independent experiments. Standard errors are shown. (B) Cell population doubling time (PDT) analysis of mid–late passage VSMCs treated with DMSO, 1 or 0.1 μm Remodelin. No differences were detected between DMSO and 0.1 μm Remodelin‐treated cells, but 1 μm Remodelin‐treated cells retained low PDTs for longer and grew significantly faster at passages 19, 21 and 23 (*P < 0.05, **P < 0.01). Data were from 3 independent experiments. (C) (Left) Representative image of senescence‐associated β‐galactosidase assay of p23 VSMCs treated with DMSO or 1 μm Remodelin from PDT experiment shown in B. Senescent cells are indicated by blue staining. (Right) Quantification of senescent cells. A minimum of 200 cells were counted from 3 experiments. (D) Cell vitality analysis of p11 VSMCs expressing EGFP, UCLA or UCLA+Remodelin. As a positive control for apoptosis initiation, cells were treated with Staurosporine. (Left) Representative plots from assay. (Right) Quantification of healthy cells from 3 independent experiments. (E) Enumeration of γH2AX and 53BP1 foci in p8 and p14 VSMCs treated with etoposide and −/+ FTI or Remodelin. Counts were from >200 cells from 3 independent experiments. (F) Representative WB of cytoplasmic (C) and nuclear (N) fractions from p10 VSMCs expressing EGFP or UCLA and treated with DMSO, FTIs or Remodelin. Prelamin A expression reduces levels of nuclear Ran, and this can be recovered to an extent by Remodelin. (G) Quantification of Ran band intensities shown in G. Measurements were taken from 3 separate experiments. Standard errors are shown. Bars in red indicate additional data added to data presented in Fig. 3A.

Reduced γH2AX in Remodelin‐treated aged VSMCs suggested genomic stability had been improved. To directly measure whether DNA damage was reduced, we used alkaline comet assays and found lower levels of DNA damage in treated late passage VSMCs (Fig. 6A), although these cells still exhibited significantly more DNA damage than low passage VSMCs.

We hypothesized that VSMCs treated with Remodelin might have extended proliferative lifespan *in vitro*. We therefore performed cell population doubling experiments alongside senescence β‐galactosidase assays to monitor proliferation and levels of senescence in mid‐to‐late passage VSMCs treated with DMSO control, or with 1 or 0.1 μm Remodelin. We found treatment with 1 μm Remodelin maintained low population doubling times compared to both DMSO and 0.1 μm Remodelin (Fig. 6B); however, treatment with 10 μm Remodelin proved to be cytotoxic over a few passages (data not shown). Analysis of senescence at passage 23 (Fig. 6C) confirmed the beneficial effect of Remodelin as there was a strong decrease in senescent VSMCs at this passage when treated with 1 μm Remodelin.

Next, we employed a cell vitality assay that measures reduced thiols to determine cell health. We expressed UCLA in young VSMCs and treated these with either DMSO or Remodelin. When compared to VSMCs expressing EGFP, UCLA expression significantly reduced cell health and many cells appeared to be at early stages of apoptosis (Fig. 6D). However, Remodelin dramatically improved cell health to similar levels as EGFP‐treated cells. To confirm that the observed improvements in cell health were related to restored 53BP1 functionality, we analysed the effect of Remodelin upon 53BP1 foci formation and found recruitment to sites was significantly improved in passage 14 VSMCs following Remodelin treatment (Fig. 6E and Fig. S7). As defects in 53BP1 recruitment had been shown to be a consequence of NUP153‐mediated disruption of the Ran gradient, we next wanted to establish whether Remodelin had restored nuclear levels of Ran in VSMCs expressing prelamin A. As shown in Fig. 6F,G, Ran nuclear compartmentalization had been restored to levels observed in controls.

## Discussion

### Prelamin A inhibits 53BP1 recruitment in aged VSMCs by deregulation of NUP153

In this present study, we demonstrated defective accumulation of 53BP1 at sites of DNA damage in aged human VSMCs that express prelamin A, despite normal phosphorylation of H2AX. Aged VSMCs also displayed an increased level of cytoplasmic 53BP1 despite no changes in overall protein level, suggesting its transport into the nucleus had been reduced. Further investigations identified that nucleoporin NUP153, a key regulator of 53BP1 nuclear import, was disorganized upon prelamin A expression in VSMCs, suggesting these alterations would impede import of 53BP1 and compromise its recruitment to DNA damage. This would explain why later 53BP1 accruement to DNA damage was more affected than early recruitment as the existing pool of nuclear 53BP1 in these cells would initially be able to respond but essential further aggregation of 53BP1 would be hindered by reduced nuclear 53BP1 levels due to decreased nuclear import. As similar observations were seen in U2OS cells, we speculate that prelamin A will impair genome stability through comparable mechanisms in different cell types including HGPS fibroblasts and other models of prelamin A accumulation where impaired recruitment of 53BP1 has been documented (Liu *et al*., 2005). It is worth noting that our data are different from previous studies that have shown depletion of A‐type lamins affects 53BP1 stability by accelerated degradation (Gonzalez‐Suarez *et al*., 2011). Despite also compromising nuclear lamina integrity, the accumulation of prelamin A is a distinctly different event to loss of lamin A protein as the mature lamina is still intact but instead, a nonmature lamin A is present. As shown by pull‐down assays, prelamin A is likely to compete against the mature lamina for NUP153 binding and alter its function due to its different biochemical properties.

The intranuclear aggregation of NUP153 we observed occurred concomitantly with a loss of this nucleoporin at the NE. Previous studies in mouse embryonic fibroblasts have shown that these aggregates are concentrated in intranuclear invaginations collectively termed the nuclear reticulum (NR) (Goulbourne *et al*., 2011). Previously, the trapping of NUP153 in NR was proposed to arise as an adaptive response to the build‐up of toxic prelamin A to facilitate mRNA export by shortening the distance between intranuclear expressing genes and the cytoplasm (Goulbourne *et al*., 2011). Instead, we propose that it is the prelamin A‐mediated formation of the NR and subsequent trapping of this key nucleoporin that is the toxic event, as cytoplasmic‐nuclear trafficking is compromised. We did not detect changes in positioning of all nucleoporins; notably, NUP62 was retained at the NE. The position of NUP153 close to the INM where prelamin A localizes may explain why this nucleoporin is particularly affected. How mislocalized NUP153 reduces nuclear Ran levels remains to be understood; however, disruption of the nuclear basket may be a key factor.

The accumulation of prelamin A is not the only change that can occur to the nuclear lamina of aging cells and it is possible that other factors could contribute to NUP153 mislocalization in late passage VSMCs. However, as we observed identical results by expressing prelamin A in U2OS cells that would otherwise have normal nuclear lamina, our hypothesis that prelamin A is the key determinant in NUP153 mislocalization in aged VSMCs is strengthened.

### Prelamin A‐induced mislocalization of NUP153 deregulates the Ran gradient

NUP153 has been shown to be directly involved in 53BP1 nuclear import (Lemaitre *et al*., 2012), but the precise mechanisms responsible for this remain unknown. We hypothesized that mislocalization of NUP153 may affect the Ran gradient by interfering with its import into the nucleus, potentially by interrupting interactions between its key nuclear import regulator NTF2 (nuclear transport factor 2) and the NPC (Ribbeck *et al*., 1998). As production of the RanGTP gradient relies heavily on establishment of a Ran protein gradient (approximately 3 times more nuclear Ran than cytoplasmic), this would in turn compromise the RanGTP gradient and thereby reduce nuclear import of large proteins such as 53BP1 in cells containing prelamin A. We confirmed prelamin A expression compromised nuclear import of Ran in VSMCs, and this impaired import was recapitulated in cells depleted of NUP153. These data link for the first time NUP153 in this process and shed light on the underlying mechanism for why nonmature lamin A affects the Ran gradient. These results are also consistent with studies implicating progerin in the abrogation of the Ran gradient and subsequent decrease of large cargo nuclear import (Kelley *et al*., 2011; Snow *et al*., 2013). How NUP153 impedes import of Ran protein remains unclear and merits further investigation; however, a link between deficient Ubc9‐mediated SUMOylation and Ran protein nuclear import has been proposed (Kelley *et al*., 2011; Datta *et al*., 2014).

### Remodelin alleviates defects associated with prelamin A and improves cell health

We have shown that prelamin A found in aged VSMCs is farnesylated. As inhibitors of farnesyltransferases have long been seen as a potential therapy to alleviate defects of prelamin A or progerin expression, we tested the effects of FTIs on aged VSMCs. Despite successfully reducing farnesylated species of prelamin A and subsequently releasing its tight association with the NE and improving nuclear circularity, genomic stability was not improved. In contrast, we found that a newly reported inhibitor of NAT10 acetyltransferase, termed Remodelin, was able to delay senescence and maintain low cell population times at later passages alongside improving many prelamin A‐induced abnormalities, including nuclear blebbing, NUP153 disorganization, genomic instability and 53BP1 cytoplasmic accumulation. These findings highlight the utility of this compound as a tool to restore cellular homeostasis in affected cells and also demonstrate the potential use of this or other NAT10 inhibitors as therapeutic agents. Whilst the precise mechanisms that underlie the benefits of NAT10 inhibition remain unclear, changes to microtubule anchorage that occur following reduced NAT10 activity may allay additional forces that have been put upon the nucleus following alterations to the nuclear lamina (Larrieu *et al*., 2014). In the model we are proposing, this relaxing of external forces upon the nucleus would cause a reduction in NR formation, release of trapped NUP153, restoration of the Ran gradient and improved trafficking between the nucleus and cytoplasm. For this, an intact and functional microtubule network is likely to be essential for Remodelin to effectuate its benefits and allow for unfavourable changes in the NE to be reversed.

## Conclusions

We have shown that aged VSMCs with accumulated prelamin A display aberrant NUP153 localization, and provide evidence that this, in turn, impedes 53BP1 entry into the nucleus and restricts its access to sites of DNA damage. This effect is likely to prevent efficient DSB repair and may be responsible for the elevated levels of unrepaired DNA damage observed in aged VSMCs. We also show that NUP153 mislocalization impedes the Ran protein gradient and that this may explain how defects in NUP153 affect import of 53BP1. Additionally, we have shown Remodelin is able to reverse many prelamin A‐dependent abnormalities in VSMCs and thus may have therapeutic potential in the context of cardiovascular disease.

## Experimental procedures

Experimental procedures are described in detail in the supplements.

## Conflict of interest

The authors declare that they have no conflict of interests.

## Supporting information


**Fig. S1** Late passage VSMCs accumulate prelamin A in culture.
**Fig. S2** Overexpression of prelamin A but not wild‐type lamin A attenuates 53BP1 recruitment to DNA damage.
**Fig. S3** Expression of prelamin A in U2OS cells induces similar defects as aged VSMCs.
**Fig. S4** Histone marks associated with 53BP1 recruitment are not affected by prelamin A expression.
**Fig. S5** Prelamin A induces cytoplasmic accumulation in U2OS cells.
**Fig. S6** Remodelin reverses prelamin A‐dependent defects in U2OS cells.
**Fig. S7** Representative image of γH2AX and 53BP1 foci formation in VSMCs treated with FTIs and Remodelin.

**Appendix S1** Materials and methods.
