# Supplementary figures and images for: Prelamin A impairs 53BP1 nuclear entry by mislocalizing NUP153 and disrupting the Ran gradient
Source: Aging Cell. 2016 Jul 27;15(6):1039–50. doi: 10.1111/acel.12506 (PMC5114580; doi:10.1111/acel.12506)

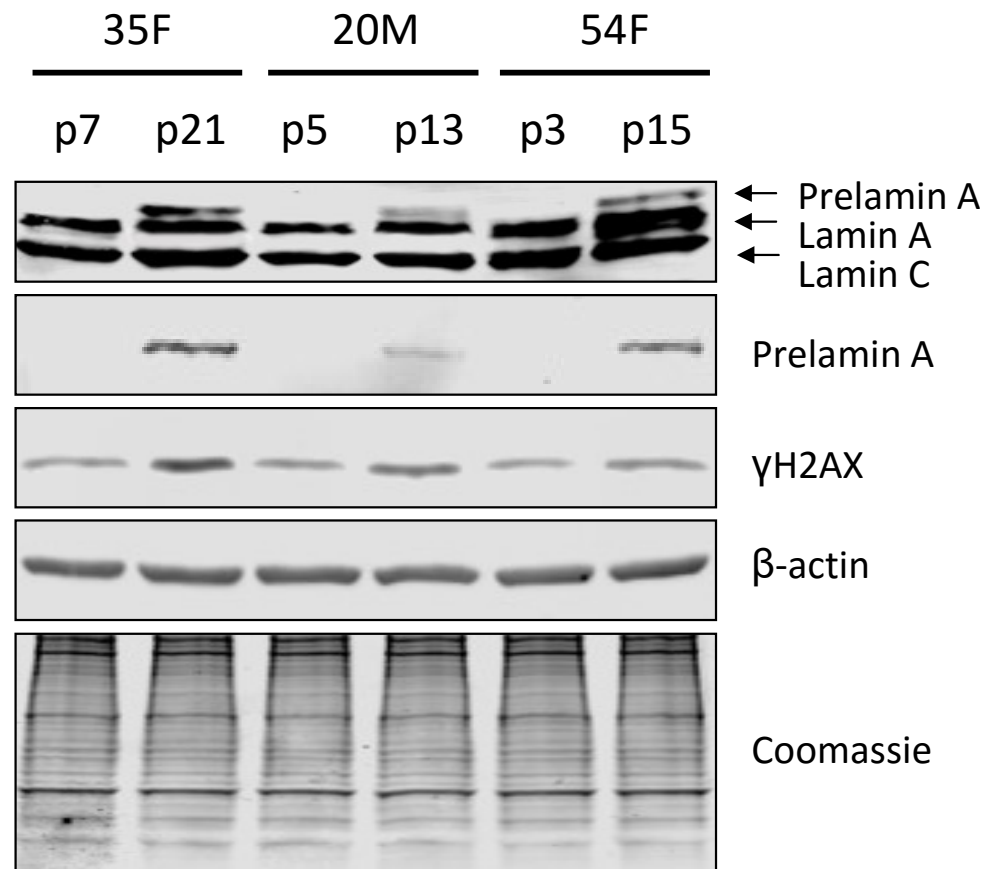

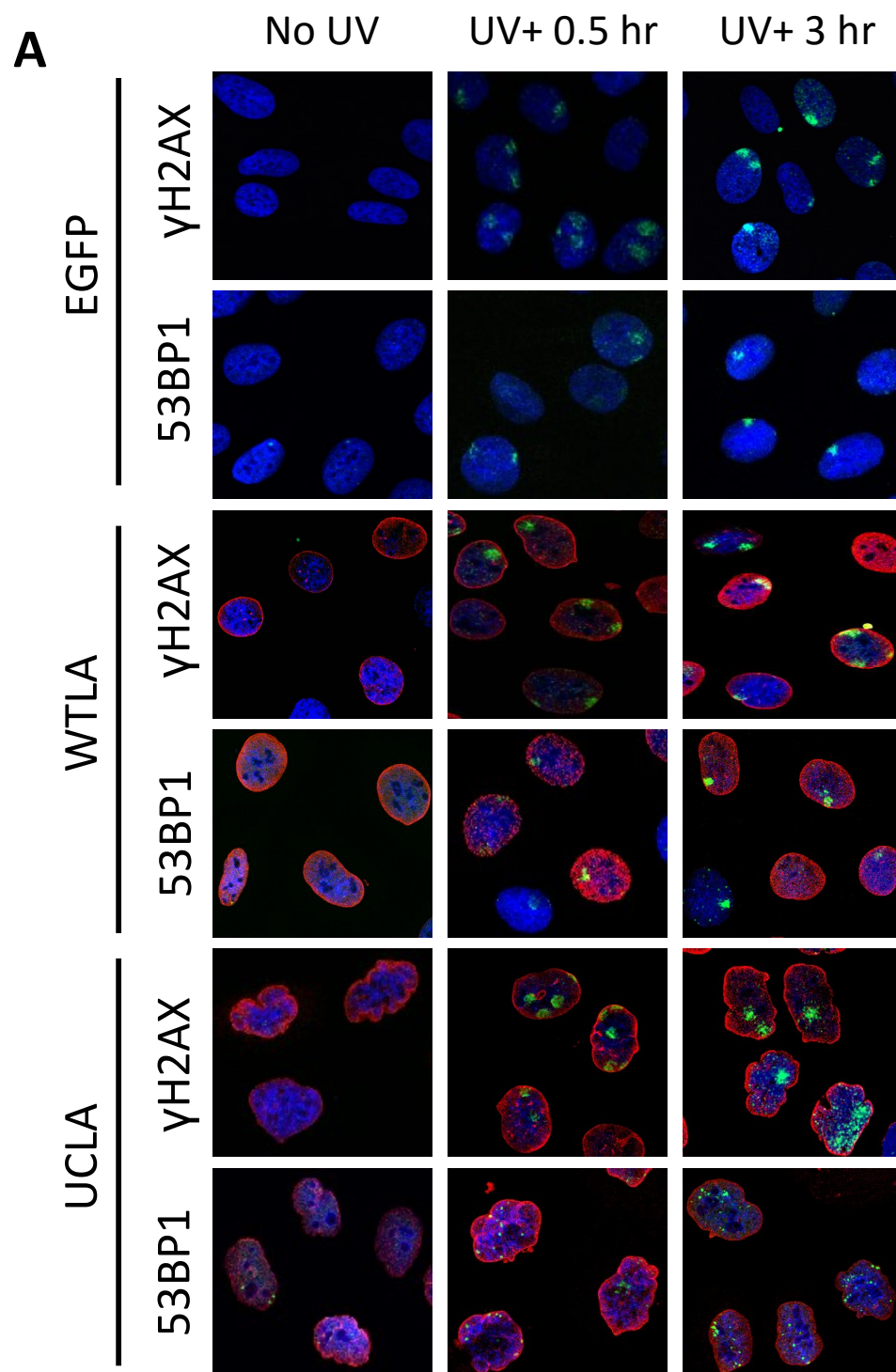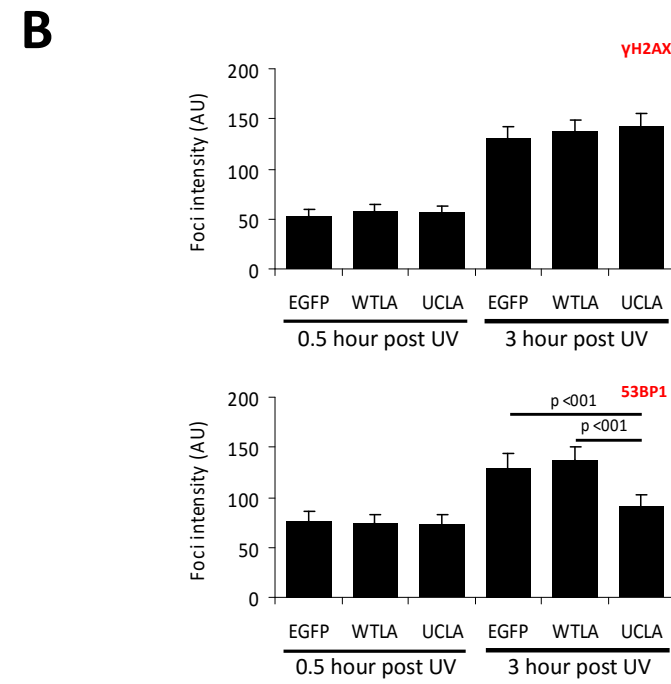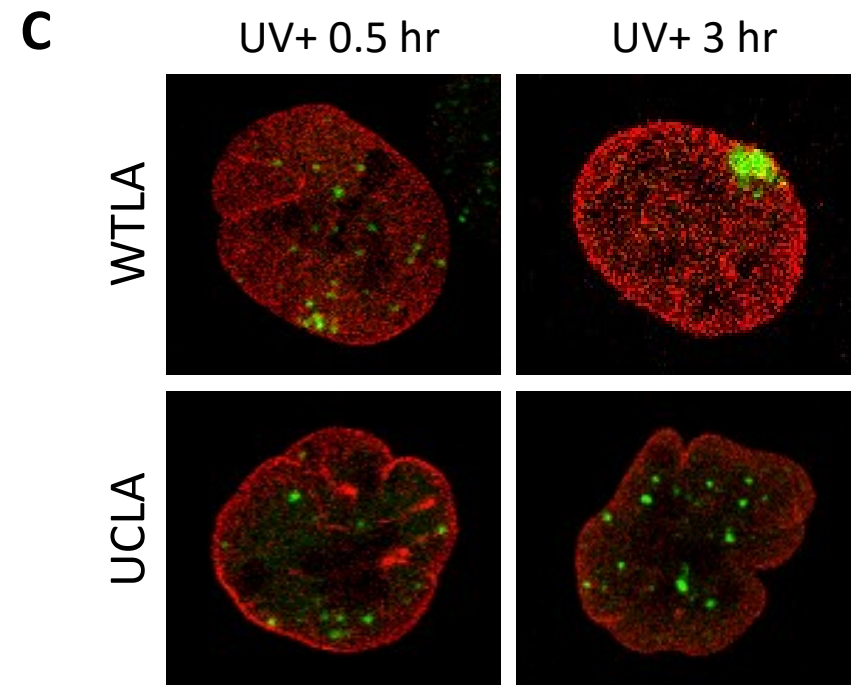

**A**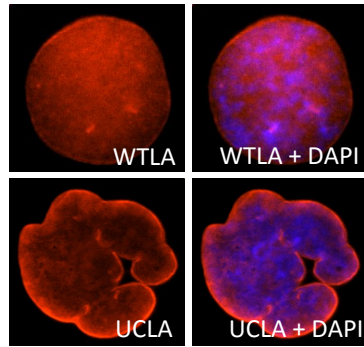**B**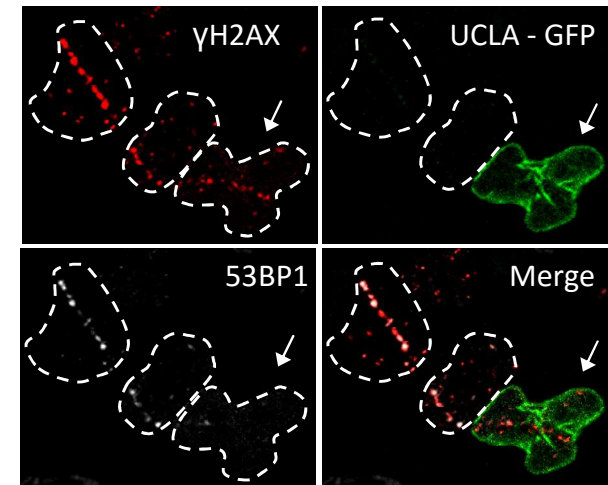**C**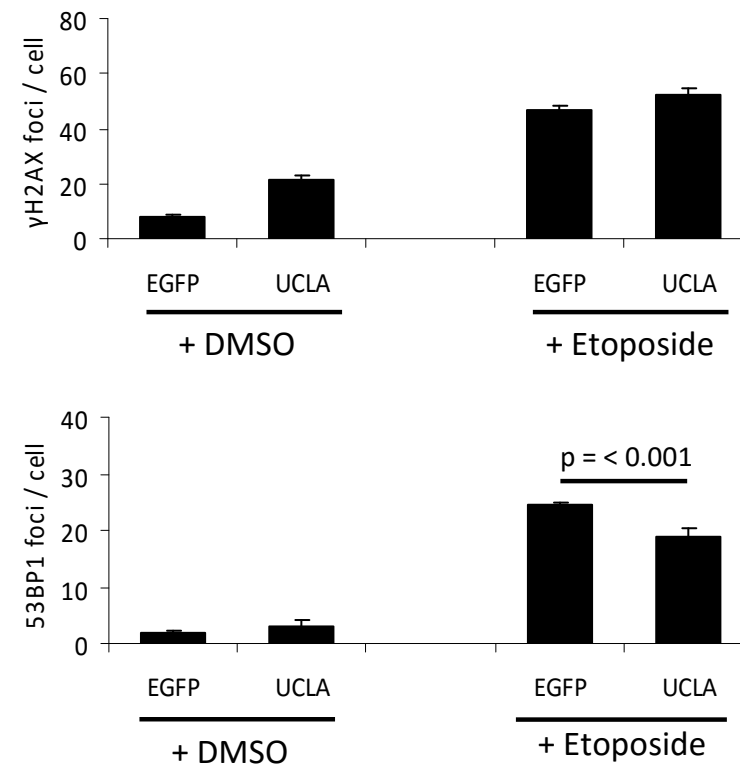

**A**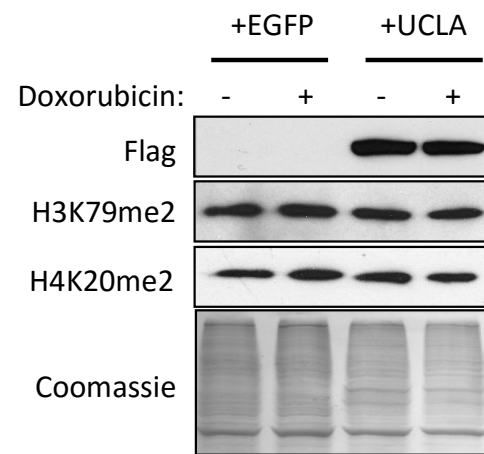**B**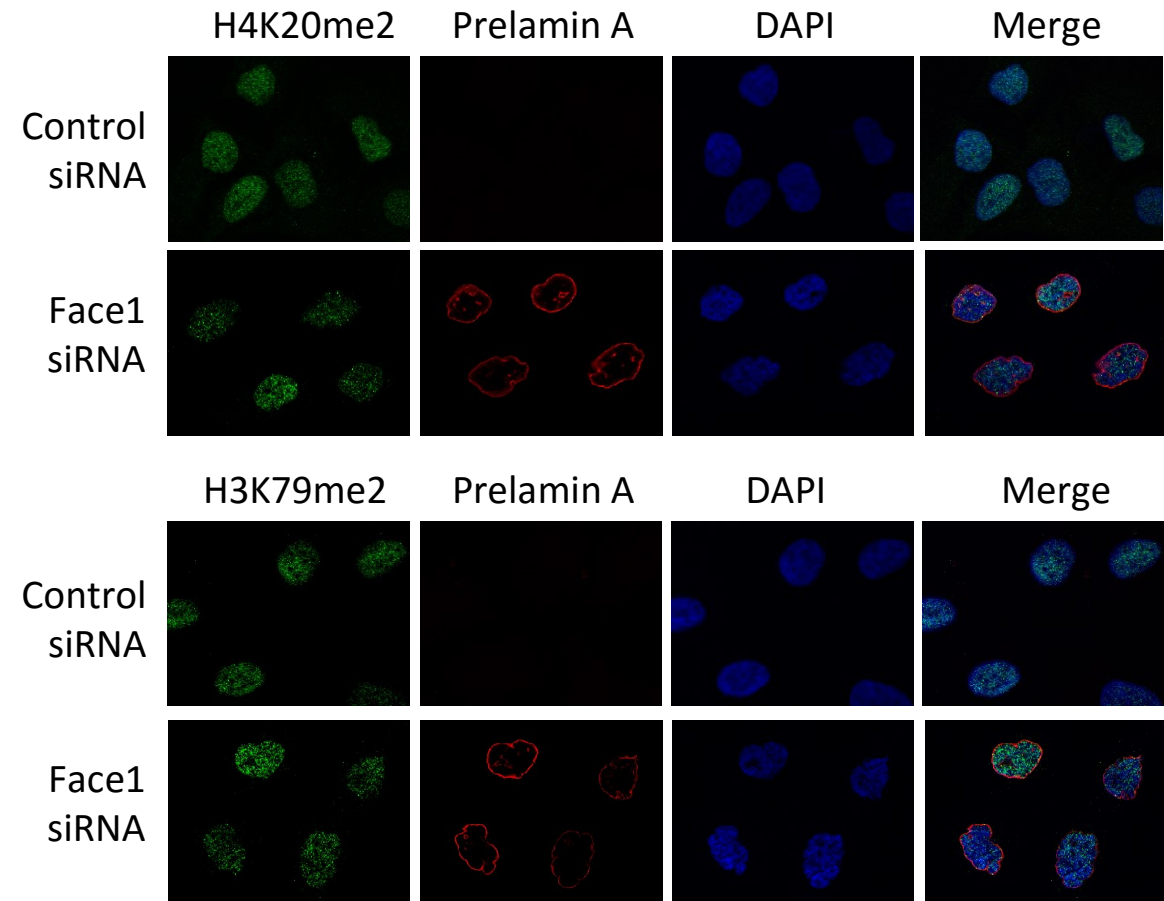

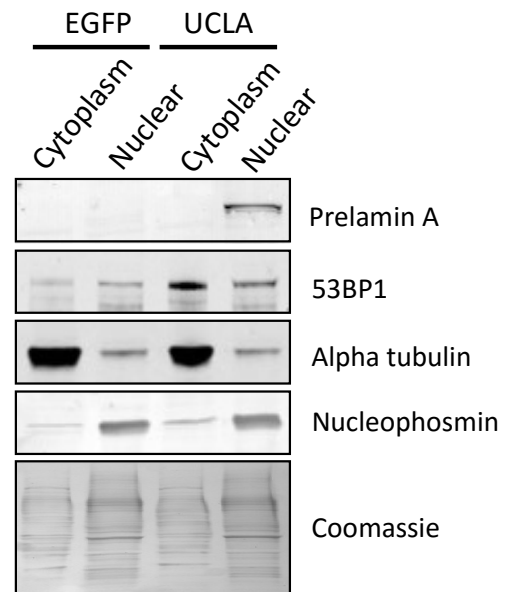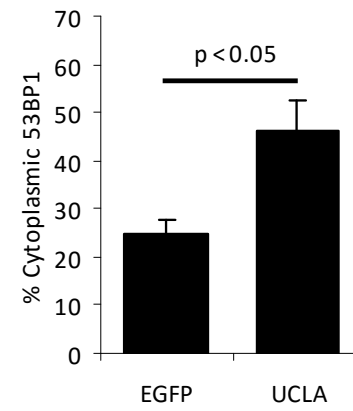

**A**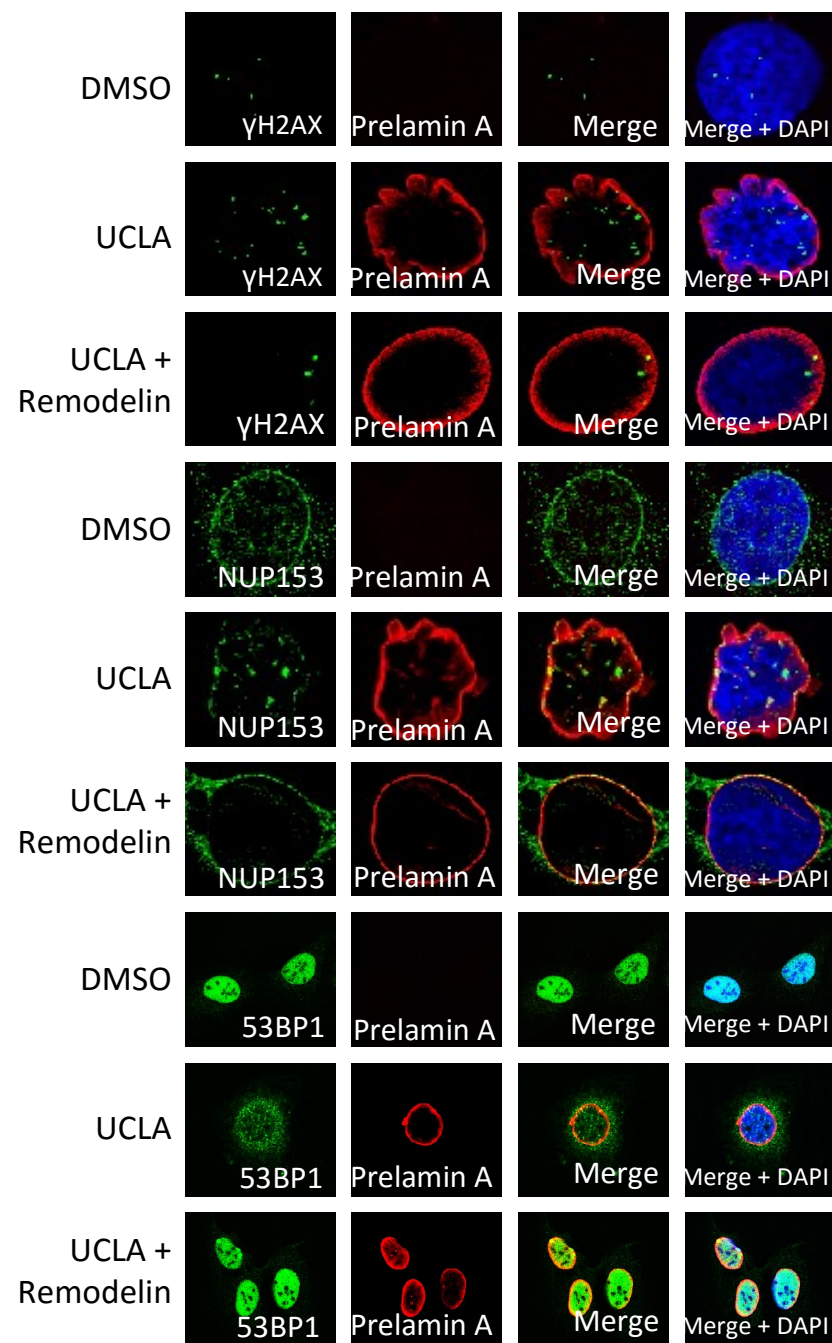**B**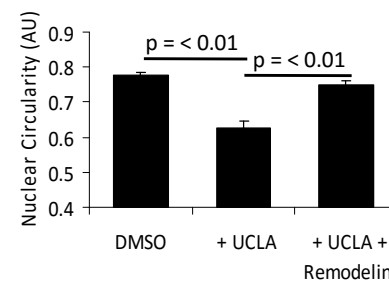**C**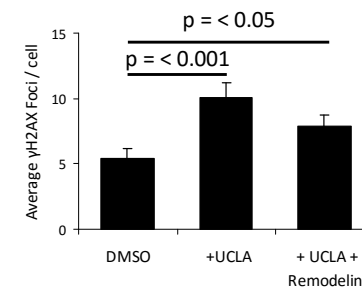**D**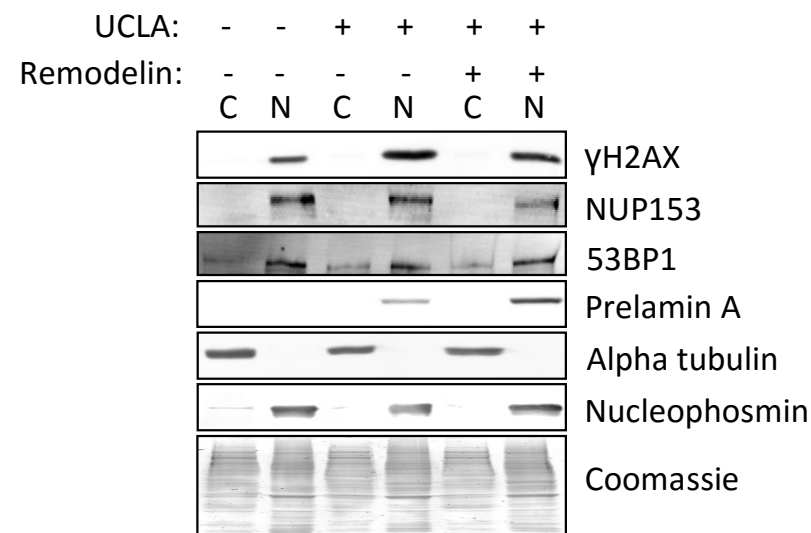**E**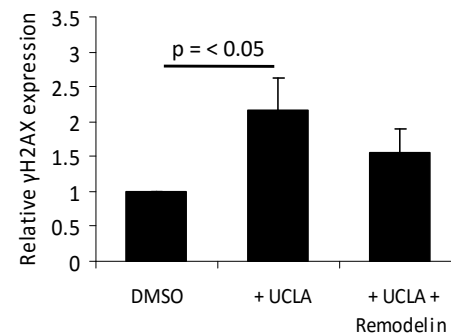**F**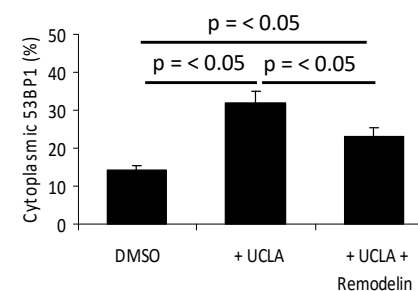

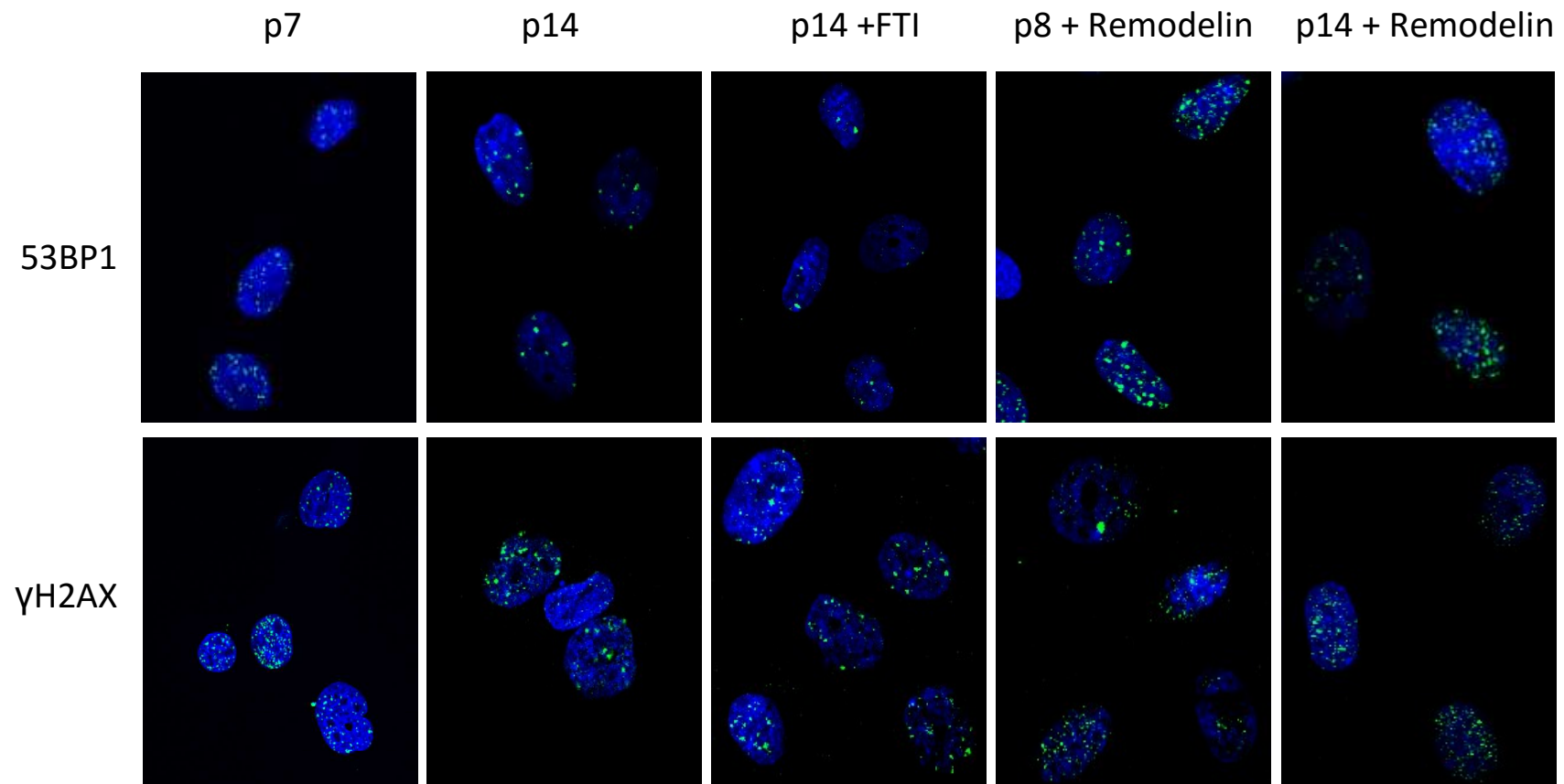

Supplement: Supplementary file 1 — Fig. S1 Late passage VSMCs accumulate prelamin A in culture. Fig. S2 Overexpression of prelamin A but not wild‐type lamin A attenuates 53BP1 recruitment to DNA damage. Fig. S3 Expression of prelamin A in U2OS cells induces similar defects as aged VSMCs. Fig. S4 Histone marks associated with 53BP1 recruitment are not affected by prelamin A expression. Fig. S5 Prelamin A induces cytoplasmic accumulation in U2OS cells. Fig. S6 Remodelin reverses prelamin A‐dependent defects in U2OS cells. Fig. S7 Representative image of γH2AX and 53BP1 foci formation in VSMCs treated with FTIs and Remodelin. [file ACEL-15-1039-s001.pdf]
