# Supplementary material for: Prelamin A impairs 53BP1 nuclear entry by mislocalizing NUP153 and disrupting the Ran gradient
Source: Aging Cell. 2016 Jul 27;15(6):1039–50. doi: 10.1111/acel.12506 (PMC5114580; doi:10.1111/acel.12506)
Supplement: Supplementary file 2 — Appendix S1 Materials and methods. [file ACEL-15-1039-s002.doc]

**Supplementary information**

**Figure legends**

**Supplementary Figure 1 – Late passage VSMCs accumulate prelamin A in culture.**

WB of whole cell lysates from three different VSMC isolates (35F, 20M and 54F). In each isolate, prelamin A is detectable at later passage using antibodies for lamin AC or antibodies specific for prelamin A. The expression of prelamin A occurs concomitantly with increased levels of γH2AX indicating higher levels of DNA damage. Β-actin and coomassie staining are shown as loading controls.

**Supplementary Figure 2 –** **Overexpression of prelamin A but not wild-type lamin A attenuates 53BP1 recruitment to DNA damage.**

**A** IF showing VSMCs expressing EGFP, Flag-tagged mature wild-type lamin A (WTLA) or Flag-tagged prelamin A (UCLA) that had been presensitised with BrdU then irradiated with 500 J/m^2^ filtered UV and left for 0.5 or 3 hours. Cells were stained with anti-Flag (red), γH2AX and 53BP1 (both green). DAPI is also shown (blue).

**B** Quantification of **A**. Whilst all EGFP, WTLA and UCLA cells exhibited similar γH2AX staining at all time points, UCLA expressing cells had marked delayed 53BP1 recruitment 3 hours after irradiation. *n* > 100 cells from 3 experiments.

**Supplementary Figure 3 –** **Expression of prelamin A in U2OS cells induces similar defects as aged VSMCs.**

**A** IF analysis showing U2OS cell nuclei expressing mature wild-type lamin A (WTLA) or prelamin A (UCLA) (both red). Prelamin A induced similar nuclear blebbing as seen in aged VSMCs that also express prelamin A. WTLA did not cause similar changes. DAPI is also shown (blue).

**B** IF analysis of γH2AX (red) and 53BP1 (white) recruitment to microirradiation induced DSBs in U2OS cells transfected with UCLA-GFP recombinant form of prelamin A (green) (indicated by white arrows). Cells were fixed 3 hours after irradiation.

**C** γH2AX and 53BP1 foci enumeration from immunofluorescence analysis of U2OS cells expressing EGFP or UCLA and treated with DMSO or etoposide. Standard errors are shown.

**Supplementary Figure 4 – Histone marks associated with 53BP1 recruitment are not affected by prelamin A expression.**

**A** WB showing expression of Flag-tagged UCLA in U2OS cells does not affect H3K79me2 or H4K20me2 protein levels. The induction of DNA damage (etoposide) also does not affect protein levels of these modified histones.

**B** IF analysis of the spatial arrangement of H4K20me2 (green) in early passage (p10) and late passage (p18) VSMCs. Cells were also treated with doxorubicin to identify if rearrangements occurred following DNA damage induction.

**Supplementary Figure 5 – Prelamin A induces cytoplasmic accumulation in U2OS cells.**

(**Left**) WB of cytoplasmic and nuclear fractions of U2OS cells expressing EGFP or UCLA. Alpha tubulin, nucleophosmin and coomassie staining are shown as loading controls. (**Right**) Quantification of 53BP1 band intensities shown in **D**. The results show expression of prelamin A causes a significant shift of 53BP1 into the cytoplasm from the nucleus. Standard errors are shown.

**Supplementary Figure 6 –** **Remodelin reverses prelamin A-dependent defects in U2OS cells.**

**A** IF analysis of U2OS cells treated with DMSO (control), UCLA (prelamin A expression) and UCLA alongside Remodelin. Cells were stained for γH2AX, NUP153 or 53BP1 (all green), prelamin A (red) and DAPI. Cells showing 53BP1 staining are at a lower magnification to allow visualisation of cytoplasmic regions.

**B** Quantification of nuclear circularity of U2OS cells expressing prelamin A with or without Remodelin treatment. Cells were visualised using IF and nuclear circularities calculated using ImageJ. Values closer to 1 indicate nuclei that are more circular. Standard errors are shown.

**C** Enumeration of γH2AX foci in control U2OS cells and U2OS cells expressing prelamin A with or without Remodelin treatment following IF analysis. Standard errors are shown.

**D** WB analysing the affect of Remodelin upon U2OS cells expressing prelamin A (UCLA). Cytoplasmic (C) and nuclear (N) lysates from control or UCLA expressing U2OS cells (with and without Remodelin treatment) were analysed. Remodelin treatment of prelamin A expressing cells reduced levels of γH2AX and alleviated the cytoplasmic accumulation of 53BP1 seen in untreated prelamin A positive cells despite prelamin A still being present. Alpha tubulin (cytoplasmic), nucleophosmin (nuclear) and coomassie are shown as loading controls.

**E** γH2AX band intensities were measured from 3 separate experiments shown in **D**. Standard errors are shown.

**F** 53BP1 band intensities were measured from 3 separate experiments shown in **D** and values of % cytoplasmic 53BP1 were calculated. Standard errors are shown.

**Supplementary Figure 7 –** **Representative image of γH2AX and 53BP1 foci formation in VSMCs treated with FTIs and Remodelin.**

53BP1 and γH2AX (both green) were enumerated in control p8 and p14 VSMCs, p14 VSMCs treated with FTIs and p8 and p14 VSMCs treated with Remodelin. DAPI is also stained (blue).

**Materials and methods**

**Cell culture / DNA damage induction / Drug treatments**

Human VSMCs were cultured from explants as described previously . Osteosarcoma cells (U2OS) were obtained from American Tissue Culture Collection. Farnesyl transferase inhibitor FTI-276 (R&D Systems, Minneapollis, USA) was used at 25 µM. Remodelin was used at 1 or 10 µM, typically for 3 days. For DNA damage induction, cells were treated for 3 hours with either 1 µM doxorubicin or 1 µM etoposide unless otherwise stated. UV irradiation was performed using a UV Stratalinker® 1800 (Stratagene®,California, USA) on cells presensitised with 10 µM BrdU for 24 hours with a dose of 40 J/m^2^. Laser micro-irradiation was performed as previously described . Staurosporine (Sigma) was used at 1 µM for 6 hours during cell vitality assays. Importazole (Sigma) was used at 40 µM for 24 hours to block β-importin mediated nuclear import.

**Antibodies and immunofluorescence**

Primary antibodies were sourced as follows: prelamin A (SC-6214, C-20), lamin A/C (SC-6215, N-18), GAPDH (SC-25778), Face1 (SC-34777), β-actin (SC-4778, C-4), p21 (SC-397, C-19) (Santa Cruz Biotechnology, Inc, Santa Cruz, California, USA); γ-H2AX (ab26350), Nucleophosmin (ab10530), Sun2 (ab124916), NUP153 (ab4872), NUP62 (ab96134), NPC (ab73291), Ubc9 (ab33044), Ran (ab53775), RNF8 (ab15850), Alpha tubulin (ab18251), TPR (ab58344) (abcam, Cambridge, UK); FLAG (M2, F3165) (Sigma, St. Louis, USA); γ-H2AX (2577), 53BP1 (4937), H3K79me2 (5427), H4K20me2 (9759), PCNA (PC10) (Cell Signaling Technology, Danvers, USA); 53BP1 (NB100-304) (Novus Biologicals); Anti-cleaved farnesylated prelamin A (ANT0046) (Diatheva, Fano, Italy); NAT10 (13365-1-AP) (ProteinTech, Chicago, USA). Immunofluorescence was performed as previously described .

**Adenoviral constructs and transfections**

VSMCs or U2OS cells at 70% to 80% confluence were infected with FLAG-tagged recombinant adenoviruses containing either mature wild-type lamin A (WTLA) or an uncleavable form of prelamin A mutated within the Zmpste24/Face1 cleavage site (L647R) (UCLA), or EGFP control (EGFP). Multiplicity of infection was 5 particles per cell, routinely achieving >80% transduction efficiency as assessed by the control EGFP.

For transfections, 2 µg of plasmid (either uncleavable lamin A (L647R) in shuttle vector (UCLA-GFP) or shuttle vector alone (control) was added to serum free Optimem (Gibco Invitrogen) and Superfect (Qiagen, Hilden, Germany), mixed and incubated for 10 minutes at room temperature (RT). DMEM was then added and the mix was added to cells and incubated for 2 hours at 37°C. The solution was removed, cells were washed with PBS and fresh DMEM media added.

**Cytoplasmic / Nuclear fractionations**

For separation of cytoplasmic and nuclear fractions, cells were washed once with ice cold PBS, scraped into fresh PBS, centrifuged then resuspended in Nuclear fractionation buffer (10 mM HEPES pH 7.9, 10 mM KCl 0.5 mM DTT, 0.05% NP40, protease inhibitors) and incubated on ice for 30 minutes. Samples were centrifuged and supernatants (cytoplasmic fraction) were collected. Pellets were washed once, resuspended in IP buffer (10 mM Tris pH 7.5, 150 mM NaCl, 1 mM EDTA, 1% Triton X-100, protease inhibitors) and sonicated for 10 seconds prior to final centrifugation and collection of the supernatant (nuclear fraction).

**Cellular biochemical fractionations**

Approximately 2 x 10^6^ cells were washed 2x in ice cold PBS then resuspended in extraction buffer A (50 mM HEPES pH 7.5, 150 mM NaCl, 1 mM EDTA, 10% glycerol, 0.1% triton X-100, 10 mM NaF, 10 mM β-glycerophosphate, 1 mM sodium orthovanadate, protease inhibitors) and incubated on ice for 15 minutes. Cytoplasmic protein fraction was collected following centrifugation (supernatant) and the pellet was resuspended in extraction buffer B (50 mM HEPES pH 7.5, 150 mM NaCl, 1 mM EDTA, 10 mM NaF, 10 mM β-glycerophosphate, 1 mM sodium orthovanadate, protease inhibitors) and incubated for 30 minutes at RT with occasional agitation. Following centrifugation, the nuclear soluble fraction (supernatant) was collected and pellets were resuspended in extraction buffer C (50 mM HEPES pH 7.5, 150 mM NaCl, 1 mM EDTA, 10 mM NaF, 10 mM β-glycerophosphate, 1 mM sodium orthovanadate, 300 µg/ml DNase I, protease inhibitors) for 30 minutes at 37°C. Following centrifugation, supernatant was collected (chromatin-associated fraction), pellets were resuspended in PBS, heated at 100°C for 10 minutes and sonicated for 10 seconds and centrifuged to collect nuclear insoluble fraction in the supernatant.

**Small RNA–mediated interference**

Smart pool FACE1, NUP153, LMNA or control small interfering RNA (siRNA) oligonucleotides (Dharmacon, Lafayette, USA) were transfected into VSMCs or U2OS cells with the use of HiPerfect transfection reagent (Qiagen). At 72 hours after transfection, samples were prepared for cell survival analysis or western blotting.

**Flag-immunoprecipitation assays**

U2OS cells were transduced with EGFP, WTLA or UCLA as described above. Cell lysates were obtained by sonicating cells in IP buffer and collecting supernatant after centrifugation. ANTI-FLAG® M2 Affinity Gel slurry (Sigma) was added to 400 µg of protein and IP buffer was added to a final volume of 500 µl. Samples were incubated at 4°C, rotating for 2 hours. Bead-protein complexes were washed 3x in IP buffer and finally the pellet was resuspended in 4x sample buffer, heated at 100°C for 10 minutes and analysed by western blot.

**Co-immunoprecipitation assays**

VSMCs cells were harvested in RIPA buffer on ice for 30 minutes with occasional agitation followed by 5s sonication and centrifugation. 300 µg of protein was incubated with Protein A beads (Sigma) for 1 hour at 4 °C. Beads were removed by centrifugation and 1.5 µg primary antibody was added to lysates and left rotating at 4 °C for 12 hours. Following this, fresh were added to reactions and incubated for 2 hours rotating at 4 °C. Samples were then centrifuged and supernatant discarded. Pellets were washed 3x in IP buffer then finally resuspended in sample buffer, heated at 100 °C for 10 minutes and analysed by Western blot.

**Comet assays**

Assays were performed as described previously .

**Cell nuclei circularity quantification**

‘Analyse particles’ function in ImageJ was used to measure circularity of cell nuclei, with circularity values given between 0-1 (values closer to 1 being more circular in shape).

**Fluorescence intensity quantification**

Measurements were taken using ImageJ. In each region of interest, 3 measurements were taken using the Analyse measurements function. These measurements were averaged and a background reading was taken and subtracted from this value.

**Cell population doubling**

Experiments were performed by passaging VSMCs 1:2 every 2 days and the total number of cells were counted in triplicate before and after passaging. Population doubling time (PDT) was calculated using the formula T_d_=2*log(2)/log(P_2_/P_1_).

Cell senescence assays

Senescence β-Galactosidase staining kit (Cell Signaling, Danvers, USA) was used to quantify senescent cells. Assays were performed according to the manufacturers instructions.

Cell vitality assays

Assays were performed using Vitality (VB-48) protocal on a Nucleocounter NC-3000 (ChemoMetec). Experiments were performed according to the manufacturers specifications.

Statistical analyses

All data are represented as the mean ± standard error from at least three independent experiments. Statistical analysis was performed with GraphPad software with Student’s t-tests used to determine the significance of the differences between the means of individual groups. For cell and foci enumeration, statistical analysis was performed on n = 100 – 200 cells in triplicate for each control and experimental group and results were verified in a minimum of three independent experiments.

References

Liu H, Smith AJ, Lott MC, Bao Y, Bowater RP, Reddan JR , Wormstone IM (2013). Sulforaphane can protect lens cells against oxidative stress: implications for cataract prevention. *Investigative ophthalmology & visual science*. 54, 5236-5248.

Polato F, Callen E, Wong N, Faryabi R, Bunting S, Chen HT, Kozak M, Kruhlak MJ, Reczek CR, Lee WH, Ludwig T, Baer R, Feigenbaum L, Jackson S , Nussenzweig A (2014). CtIP-mediated resection is essential for viability and can operate independently of BRCA1. *The Journal of experimental medicine*. 211, 1027-1036.

Ragnauth CD, Warren DT, Liu Y, McNair R, Tajsic T, Figg N, Shroff R, Skepper J , Shanahan CM (2010). Prelamin A acts to accelerate smooth muscle cell senescence and is a novel biomarker of human vascular aging. *Circulation*. 121, 2200-2210.

Shanahan CM, Cary NR, Salisbury JR, Proudfoot D, Weissberg PL , Edmonds ME (1999). Medial localization of mineralization-regulating proteins in association with Monckeberg's sclerosis: evidence for smooth muscle cell-mediated vascular calcification. *Circulation*. 100, 2168-2176.
